# SARS-CoV-2 Detection for Diagnosis Purposes in the Setting of a Molecular Biology Research Lab

**DOI:** 10.3390/mps3030059

**Published:** 2020-08-18

**Authors:** Damien Coupeau, Nicolas Burton, Noémie Lejeune, Suzanne Loret, Astrid Petit, Srdan Pejakovic, Florian Poulain, Laura Bonil, Gabrielle Trozzi, Laetitia Wiggers, Kévin Willemart, Emmanuel André, Lies Laenen, Lize Cuypers, Marc Van Ranst, Pierre Bogaerts, Benoît Muylkens, Nicolas Albert Gillet

**Affiliations:** 1 Namur Research Institute for Life Sciences (NARILIS), Integrated Veterinary Research Unit (URVI), University of Namur, 61 rue de Bruxelles, 5000 Namur, Belgium; nicolas.burton@unamur.be (N.B.); noemie.lejeune@unamur.be (N.L.); suzanne.loret@unamur.be (S.L.); astrid.petit@unamur.be (A.P.); srdan.pejakovic@unamur.be (S.P.); poulainflorian@gmail.com (F.P.); laura-alejandra.rubiano@unamur.be (L.B.); gabrielle.trozzi@student.unamur.be (G.T.); laetitia.wiggers@unamur.be (L.W.); kevin.willemart@unamur.be (K.W.); 2 KU Leuven, Laboratory of Clinical Bacteriology and Mycology, campus Gasthuisberg, Herestraat 49, 3000 Leuven, Belgium; emmanuel.andre@uzleuven.be (E.A.); lies.laenen@uzleuven.be (L.L.); lize.cuypers@uzleuven.be (L.C.); Marc.VanRanst@uz.kuleuven.ac.be (M.V.R.); 3 Department of Laboratory Medicine, National Reference Center for Respiratory Pathogens, University Hospitals Leuven, Herestraat 49, 3000 Leuven, Belgium; 4 CHU UCL NAMUR, Laboratory of microbiology and molecular biology, Site Godinne, 5530 Yvoir, Belgium; Pierre.bogaerts@uclouvain.be

**Keywords:** SARS-CoV-2, diagnosis, COVID-19, RTqPCR

## Abstract

The emergence of the SARS-CoV-2 virus and the exponential growth of COVID-19 cases have created a major crisis for public health systems. The critical identification of contagious asymptomatic carriers requires the isolation of viral nucleic acids, reverse transcription, and amplification by PCR. However, the shortage of specific proprietary reagents or the lack of automated platforms have seriously hampered diagnostic throughput in many countries. Here, we provide a procedure for SARS-CoV-2 detection for diagnostic purposes from clinical samples in the setting of a basic research molecular biology lab. The procedure details the necessary steps for daily analysis of up to 500 clinical samples with a team composed of 12 experienced researchers. The protocol has been designed to rely on widely available reagents and devices, to cope with heterogeneous clinical specimens, to guarantee nucleic acid extraction from very scarce biological material, and to minimize the rate of false-negative results.

## 1. Introduction

Severe acute respiratory syndrome coronavirus 2 (SARS-CoV-2) is the virus responsible for the coronavirus disease of 2019 (COVID-19). The virus, first identified in Wuhan (Hubei, China) in December 2019, was declared a pandemic by the World Health Organization (WHO) in March 2020. WHO’s recommendations to mitigate the pandemic are widespread testing, quarantine of cases, contact tracing, and social distancing. Countries that have adopted and implemented these measures have so far managed to flatten their epidemic curve. However, widespread testing is not an easy task. As there are no reliable and sensitive antigenic tests available yet, the identification of infected people relies on the complex procedure of viral nucleic acid extraction followed by reverse transcription and PCR amplification. Testing is critical as asymptomatic carriers (or patients with mild symptoms) can be contagious [1]. In industrialized countries, these molecular tests are performed using automatized platforms. In an economic system based on the just-in-time rule, stocks are reduced to the bare minimum, and when the demand is global and massive (as it is for health-related products during a pandemic), a shortage is inevitable. Thus, diagnosis throughput in some industrialized and well-equipped countries is drastically reduced due to the lack of the consumables required to operate such automatized platforms. In other countries, the issues are different but the needs are similar, with the screening of health workers being a top priority.

In this extraordinary context, we describe a fast and easy-to-duplicate procedure for SARS-CoV-2 diagnosis from clinical specimens. The protocol solely relies on basic molecular biology reagents and routine devices and can be applied everywhere to deliver the urgently needed SARS-CoV-2 diagnoses.

### 1.1. Overview of the Technique

The protocol is based on the classic guanidinium thiocyanate–phenol–chloroform extraction followed by a one-step RT-qPCR. Probe and primers for SARS-CoV-2 detection were validated and published in Corman et al. (2020). By organizing the workflow of experienced technicians, PhD students or post-doc researchers, the strategy aims at providing SARS-CoV-2 diagnostic capacity everywhere in the world. Thus, one team should be able to carry out up to 500 SARS-CoV-2 tests a day. To achieve this, the team of scientists must be composed of a dozen researchers. They have to work in appropriate biosafety conditions with basic equipment. One team requires access to two class II biosafety cabinets, four laboratory chemical hoods, eight high-speed refrigerated centrifuges (14,000 *g*), and three real-time PCR instruments.

### 1.2. Applications and Target Audience

The protocol is intended for all molecular biology laboratories around the world.

### 1.3. Advantages

The protocol is independent of automated platforms and does rely only on widely available molecular biology reagents and routine devices. Further, the procedure can cope with heterogeneous clinical samples (nasopharyngeal swabs, bronchial or endotracheal aspirates or bronchoalveolar lavage fluids). Minute quantities of RNA can be confidently extracted thanks to a glycogen-based carrier. We use RNA spike-in control to minimize the rate of false-negative results. Indeed, about five percent of the clinical specimens we received show significant RT-PCR inhibition. Hence, a negative SARS-CoV-2 PCR amplification is validated as true negative only when RNA spike-in PCR performs as expected.

### 1.4. Comparison with Other Methods

Identical samples were analyzed using our protocol and using two other established procedures, respectively using the QIAamp viral RNA extraction kit (Qiagen) or the robotic extraction using Nuclisens easymag platform (Biomérieux) followed by the standard RT-qPCR as described in [2] (those kits have a limited availability). The sensitivity of the three procedures was similar. Thus, the protocol described thereafter has been approved by the Belgian federal agency for medicines and health products and by the national reference center for respiratory pathogens (university hospitals Leuven, Belgium).

### 1.5. Expertise Needed to Implement the Protocol and Limitations

The procedure is labor-intensive and requires experienced researchers.

## 2. Materials

### 2.1. Sample Reception, Deconditioning and Labeling (Pre-Analytical Step)

#### 2.1.1. Reagents

Umonium Medical Spray (Huckert’s International).

#### 2.1.2. Equipment

Class II biosafety cabinet.

#### 2.1.3. Human Resources

Two logisticians.

### 2.2. Inactivation of Viral Infectivity (Pre-Analytical Step)

#### 2.2.1. Reagents

1. Guanidinium thiocyanate solution (TRI Reagent from Sigma-Aldrich or TRIzol from Invitrogen or QIAzol from QIAGEN). CAUTION: Vapors are toxic and the solution must be handled under a chemical hood.
2. Internal Control (IC):

The IC is an RNA sequence of the Schmallenberg virus (SBV). IC RNA is stored at −80 °C.

Briefly, an 800bp-long cDNA sequence of SBV L segment has been cloned into a pGEMT plasmid (pGEMT-SBV-L1). The SBV RNA is produced by in vitro transcription using mMESSAGE mMACHINE^TM^ T7 Transcription Kit (Invitrogen) after linearization of the pGEMT-SBV-L1 by the *SpeI* enzyme. pGEMT-SBV-L1 plasmid can be provided by the UNamur URVI lab upon request (damien.coupeau@unamur.be).

3. Umonium Medical Spray (Huckert’s International).

#### 2.2.2. Equipment

Class II biosafety cabinet.

#### 2.2.3. Human Resources

Two researchers (or any person qualified to handle a molecular biology experiment).

### 2.3. RNA Extraction (Analytical Step)

#### 2.3.1. Reagents

1. Chloroform (Sigma-Aldrich). CAUTION: Vapors are toxic and the solution must be handled under a chemical hood.
2. GlycoBlue (Thermofisher Scientifc).
  Alternatively, GlycoBlue can be substituted by Glycogen, RNA grade (Thermo scientific) to serve as RNA carrier according to the manufacturer instructions.
3. Isopropanol (Sigma-Aldrich).
4. Ethanol 75% (Sigma-Aldrich). In a falcon 50 mL tube, add 12.5 mL of RNAse free water to 37.5 mL of ethanol 96–100% (Sigma-Aldrich).
5. RNAse free water.

#### 2.3.2. Equipment

1. Vortex apparatus.
2. Four to eight high-speed 1.5 mL tube refrigerated centrifuges.

#### 2.3.3. Human Resources

Eight to ten researchers (or any person qualified to handle a molecular biology experiment).

### 2.4. Taqman RT-qPCR for SARS-CoV-2 (Analytical Step)

#### 2.4.1. Reagents

1. 5X Master Mix containing DNA polymerase, MgCl_2_ (5.5 mM final concentration) and dNTPs (Eurogentec Takyon One-Step No Rox Probe 5X MasterMix dTTP). Important note: Use a low ROX or high ROX PCR Master Mix if your PCR machine requires ROX normalization.
2. Euroscript II RT (50 u/µL) and RNAse inhibitor (20 u/µL) (Euroscript II Reverse Transcriptase/RNAse inhibitor provided in the Eurogentec Takyon One-Step No Rox Probe 5X MasterMix dTTP).
3. Additive (provided in the Eurogentec Takyon One-Step No Rox Probe 5X MasterMix dTTP).
4. RNase free water.
5. Primers and Probe Mixes:

SARS-CoV-2 primers and probe (published in [2]):

E_Sarbeco_Fw: 5′- ACAGGTACGTTAATAGTTAATAGCGT-3′
E_Sarbeco_Rev: 5′- ATATTGCAGCAGTACGCACACA-3′
E_Sarbeco_Probe: 5′-(FAM)ACACTAGCCATCCTTACTGCGCTTCG(BHQ1)-3′

Internal Control primers and probe (published in [3]):

IC_Fw: 5′-TTGCCGTTTGATTTTGAAGTTGTG-3′
IC_Rev: 5′-TCAGGGATCGCAAATTAAAGAACC-3′
IC_Probe: 5′-(FAM)TCATCCGTGCTGACCCTCTGCGAG(BHQ1)-3′

Prepare Primers and Probe Mixes by mixing in a 1.5 mL tube:

Primers and Probe Mix for SARS-CoV-2
10 µL of E_Sarbeco_Probe (100 µM)
+20 µL of E_Sarbeco_Fw (100 µM)
+20 µL of E_Sarbeco_Rev (100 µM)
+950 µL of RNAse free water

Primers and Probe Mix for IC (SBV)

+10 µL of IC_Probe (100 µM)
+20 µL of IC_Fw (100 µM)
+20 µL of IC_Rev (100 µM)
+950 µL of RNAse free water

Primers and Probe Mix (5X mix) contains primers at 2 µM and probe at 1 µM.

6. Positive Controls (PC):

Heat inactivated virus stock (diluted a 10,000 times) of SARS-CoV-2-infected Vero cells was provided by the UZ/KU Leuven national reference center. RNA was extracted using classic guanidinium thiocyanate–phenol–chloroform extraction (as for the clinical specimens). Briefly, RNA from 100 µL of the diluted cell culture supernatant was extracted and resuspended in 100 µL RNAse free water. This RNA extract is thereafter called positive control 1 (PC1). Positive control 2 (PC2) is a 10-fold dilution of PC1.

Alternatively, the RNA extract of a positive clinical specimen can be used. Some positive sample generates a signal at a Ct below 20 and the RNA can, therefore, be diluted to generate a large stock of positive control.

7. Internal Control (IC), as described in Section 2.2. Inactivation of Viral Infectivity.
8. Negative Control (NC):

RNAse free water.

#### 2.4.2. Equipment

Three quantitative PCR machines (Roche Light Cycler or equivalent, make sure the PCR master mix is compatible with your machine, notably regarding ROX normalization).

#### 2.4.3. Human Resources

Three researchers (or any qualified person to handle a molecular biology experiment).

### 2.5. Data Analysis and Validation (Post-Analytical Step)

#### Human Resources

One logistician.

## 3. Procedure

### 3.1. Sample Reception, Deconditioning and Labelling (Pre-Analytical Step)

Tagged clinical specimens are shipped from the reference lab to the labs of molecular biology. If a delay in extraction is expected, store the clinical specimens at 4 °C overnight and notify the responsible at the reference center.

The logistician is responsible for the reception, deconditioning, and labeling of the clinical specimens. They connect the patient sample and its external identification tag issued by the reference lab with the internal tracking number. The internal tracking number follows the sample all over the procedure and is unique to that sample. In other words, if a hundred samples are analyzed at the first day of operation, the first sample of the second day will receive the tracking number 101.

The logistician prepares in advance a collection of labels with the tracking number written on it.

Four labels per treated sample are necessary.

1. The 1st label is stuck to the original sample;
2. The 2nd label is stuck to the sample’s sheet (medical prescription form);
3. The 3rd label is stuck to the first 1.5 mL tube for inactivation of viral infectivity;
4. The 4th label is stuck to the second 1.5 mL tube for RNA precipitation and resuspension.

The labels must be stuck on the 1.5 mL tube caps to allow visualization of the sample.

CAUTION: Upon receipt of the clinical specimens, the logistician opens the envelope containing the clinical specimen under a class II biological safety cabinet wearing appropriate protective equipment (FFP2 or FFP3 mask, safety goggles, gown and two sets of medical gloves). They stick the 1st label to the clinical specimen tube and the second to the corresponding medical prescription form. They transfer the clinical specimen tubes to the inactivation platform.

### 3.2. Inactivation of Viral Infectivity (Pre-Analytical Step)

CAUTION: Clinical specimen processing must be performed in a class II biological safety cabinet by an experienced researcher wearing appropriate protective equipment (FFP2 or FFP3 mask, safety goggles, gown and two sets of medical gloves).

**Figure 1:**
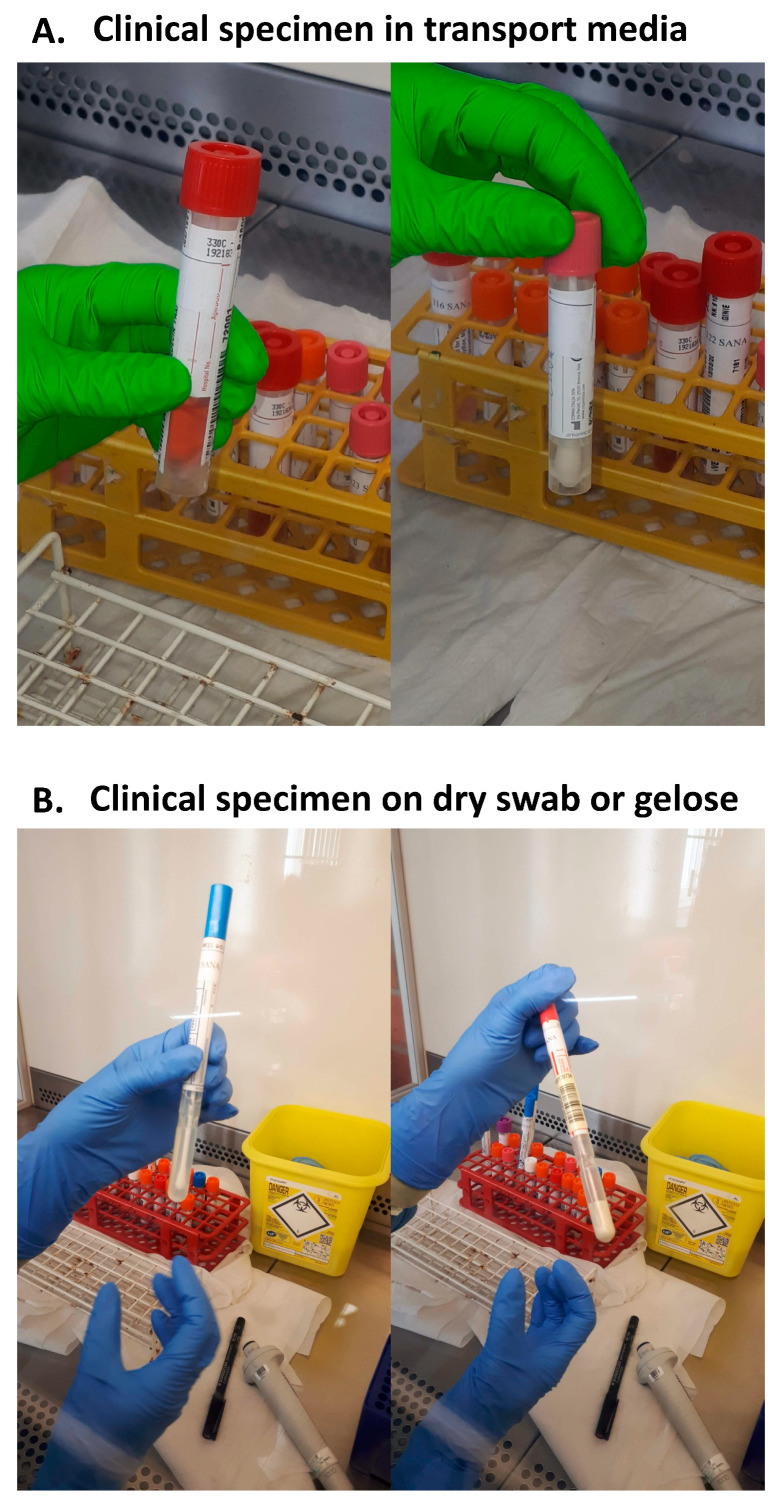
Type of clinical specimen tubes.

There are different types of clinical specimen tubes (Figure 1). In some case, the clinical specimen has been mixed with a transport solution (Figure 1A). These specimens are adequate for direct processing. In another case, the clinical specimen has been collected on a dry swab or on a swab on gel (Figure 1, panel B). These specimens are less appropriate. To analyze those specimens, the swab must be dipped in 500 µL of PBS and 100 µL should be used for RNA extraction. Keep track of specimens collected with those tubes when analyzing the data.

The whole process is carried out using filtered tips. A researcher must change filtered tips between each pipetting. A tracking form follows the samples at each steps of the protocol (Table 1).

1. Vortex nasopharyngeal swabs, aspirates or BAL (BronchoAlveolar Lavage) fluids briefly;
2. Spin the clinical specimen collection tube at 200 g for 1 min to make sure the sample (transport media inside the clinical sample collection tube) is at the bottom of the tube. Use a centrifuge with sealed buckets. Clean the centrifuge with Umonium Medical Spray or equivalent antiviral disinfectant;
3. Under a BSL2 hood, homogenize the sample by up and down pipetting and transfer 100 µL of the sample in the corresponding labeled 1.5 mL tube containing 1 mL of guanidinium thiocyanate solution (Trizol or equivalent) supplemented with 5 µL of Internal Control. Mix by inverting the tube. This procedure instantly inactivates viral infectivity;
4. The 1.5 mL tubes containing 1 mL of guanidinium thiocyanate solution supplemented with 5 µL of Internal Control must be prepared ahead under a chemical hood (vapors of guanidinium thiocyanate solution are toxic). These aliquots can be stored at −80 °C if necessary;
5. Before the samples can get out of the biosafety cabinet, spray the tubes with quaternary ammonium solution (Umonium Medical Spray or equivalent). Wipe the tubes;
6. Samples are now handled without any biosafety issue. Organize the inactivated samples by a series of 21 samples +1 tube called Extraction Control or EC. The EC tube contains 1 mL of guanidinium thiocyanate solution supplemented with 100 µL of PBS and 5 µL of Internal Control ONLY, i.e., without clinical sample. RNA extraction must be performed by a batch of 22 tubes (21 samples +1 EC);
7. If necessary, samples can be stored at −80 °C for further processing. Because we usually receive the clinical specimens by the afternoon, the deconditioning and viral inactivation steps occur at day zero and the inactivated samples are stored at −80 °C overnight. RNA extraction, RT-qPCR, and data validation and results communication occur at day +1.

### 3.3. RNA Extraction (Analytical Step)

Inactivated samples (identified by their unique tracking numbers) are treated in parallel by a team of experienced researchers or lab technicians. One researcher is typically able to handle two to three batches of 21 samples in one day. To reach 500 extractions a day, a team should be composed of eight researchers working under four chemical hoods (hood large enough to host two researchers).

The whole process is carried out using filtered tips. This step is prone to cross-contamination between samples and might lead to false-positive results. Thus, a researcher must change of filtered tips between each pipetting. Clean the chemical hood and pipettes between each batch of extraction. For each extraction batch, keep track of the operator name and hood used.

Prepare ahead 21 1.5 mL microcentrifuge tubes with the corresponding sample labels + 1 Eppendorf tube for the IC only (RNA spike-in without clinical sample).

In a 5 mL Eppendorf tube, prepare 4.8 mL of chloroform (use chloroform resistant plastic).

In a Falcon 15 mL tube, prepare 12 mL of isopropanol.

In a Falcon 50 mL tube, prepare 24 mL of 75% ethanol.

In a 1.5 mL tube, prepare 720 μL of RNAse free water.

It is critical to prepare these aliquots in a dedicated room with dedicated pipettes. Clinical specimen, RNA extract, and, of course, PCR product must never be introduced in that room.

Just before starting RNA extraction, supplement the 12 mL of isopropanol with 72 µL of GlycoBlue.

**Figure 2:**
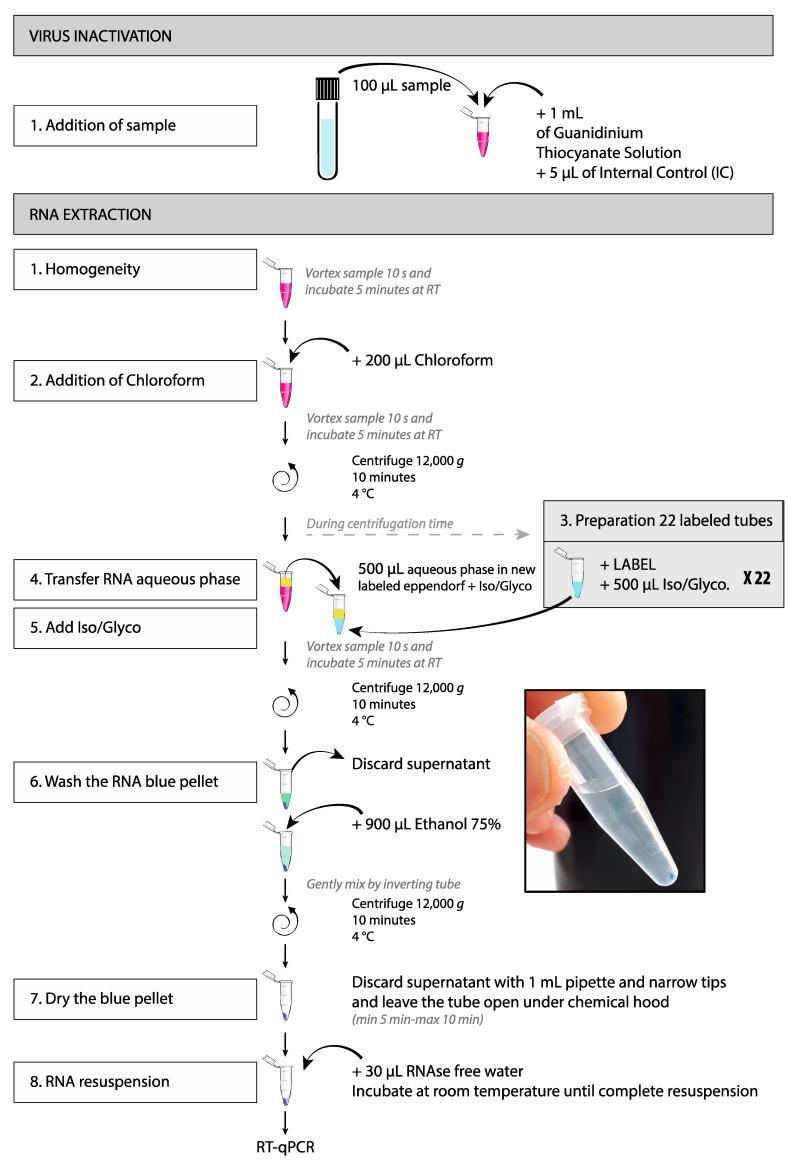
RNA extraction workflow.

To process a batch of 22 tubes (21 samples + 1 EC) (Work-flow in Figure 2):

1. Vortex the samples 10 s and incubate 5 min at room temperature;
2. Add 200 µL of chloroform and vortex for 10 s CAUTION: Vapors are toxic and the solution must be handled under a chemical hood;
3. Vortex the samples 10 s and incubate 5 min at room temperature;
4. Centrifuge at 12,000 *g* for 10 min at 4 °C;
5. Transfer 500 µL of the colorless upper aqueous phase (containing the RNA) in the second 1.5 mL tube, avoid contact with the ring or the lower organic phase (pink).

TROUBLESHOOTING: The organic and aqueous phase can be inverted, i.e., the organic pink phase can be above the clear aqueous phase. Add 100 µL of RNAse free water to the sample, vortex, centrifuge at 12,000 *g* for 10 min at 4 °C and resume from step 7).

6. Add 500 µL of the mix isopropanol-GlycoBlue;
7. Vortex for 10 s and incubate 5 min at room temperature;
8. Centrifuge at 12,000 to 14,000 *g* for 10 min at 4 °C.

The RNA pellet forms a blue pellet on the bottom of the tube (Figure 2). If no pellet is visible, restart extraction from the inactivation step.

9. Discard the supernatant;
10. Add 900 µL of 75% ethanol;
11. Gently mix by inverting the tubes;
12. Centrifuge at 12,000 to 14,000 *g* for 10 min at 4 °C;
13. Aspirate slowly the supernatant with a 1 mL pipette by avoiding contact with the blue pellet (slow pipetting allows the ethanol to drain along the tube wall);
14. Use a narrow tip (e.g., gel loading tip) to remove residual ethanol;
15. To dry the pellet, leave the tube open under the chemical hood until complete ethanol evaporation. This step lasts about 5 min. Do not over-dry the RNA by letting the sample dries more than 10 min;
16. Resuspend the pellet in 30 µL of RNAse free water;
17. Incubate at room temperature until complete resuspension of the blue pellet;
18. If necessary, RNA can be stored at −80 °C for further processing.

### 3.4. Taqman RT-qPCR for SARS-CoV-2 (Analytical Step)

The RNA samples (identified by their unique tracking numbers) are shipped to the molecular biology platform for RT-qPCR analysis. To analyze 504 samples, the team should count three researchers each running four plates (each plate allows the analysis of 42 samples).

The whole process is carried out using filtered tips. A researcher must change filtered tips between each pipetting.

**Figure 3:**
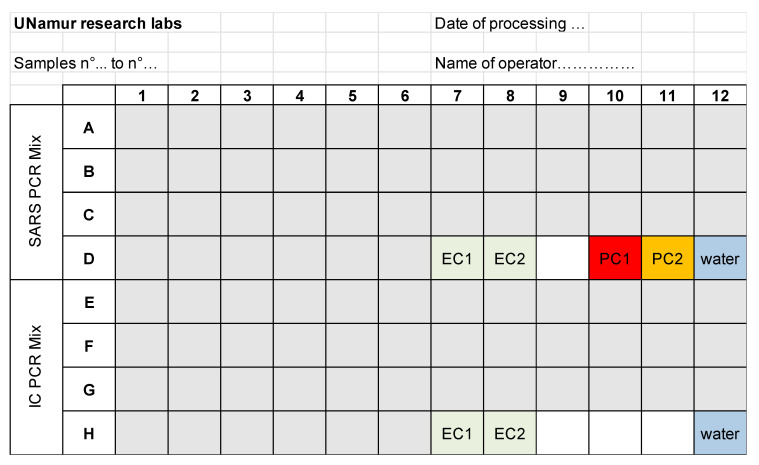
PCR loading form.

Disposition of Samples on the PCR plate (Table 2):

1. S stands for Sample, 42 samples per plate (2 batches of 21 samples);
2. NC for Negative Control (water);
3. PC1 for Positive Control 1, PC2 for Positive Control 2 (RNA from SARS-CoV-2-infected cells);
4. EC1 for Extraction Control of the first extraction batch, EC2 for Extraction Control of the second extraction batch.
5. A hard copy of the PCR plate is kept for the validation process (Figure 3).

It is critical to prepare the PCR mixes (Table 3 and Table 4) in a dedicated room with dedicated pipettes. Clinical specimen, RNA extract, and, of course, PCR product must never be introduced in that room.

1. First deposit 16 µL of the PCR Mix per well;
2. Next, add 4 µL of sample per well (or 4 µL of water for NC, or 4 µL of PC, or 4 µL of EC);
3. Stick the adhesive film;
4. Spin the PCR plate to collect the PCR mix at the bottom of the well, 200 *g*, 1 min.

Thermal protocol:

1. 48 °C 10 min;
2. 95 °C 3 min;
3. 45 cycles: 95 °C 15 s, 58 °C 30 s.

### 3.5. Data Analysis and Validation (Post-Analytical Step)

We consider the plate as VALID only if Ct values for
NC (SARS) > 45 and NC (IC) > 45
and PC1 (SARS) < 30 and PC2 (SARS) < 35.
Otherwise, the plate must be re-run.

We consider an extraction batch as VALID only if Ct value for

EC (SARS) > 45.

Otherwise, the extraction batch must be redone (EC (SARS) < 45 indicates cross-contamination and might lead to false-positive results).

We consider the sample as VALID only if

∆Ct Sample (IC) – EC (IC) < 3.3.

This allows us to exclude samples where the RT-qPCR is inhibited.

Otherwise, the original clinical specimen must be diluted 5 times in PBS and RNA must be re-extracted. Diluted samples should be marked in the tracking sheet so that the reduced sensitivity of the assay can be reported. In case of persistent inhibition, the sample is classified as “undetermined due to inhibition of the diagnostic procedure”.

For a valid sample (from a valid extraction batch and a valid plate):

The result is “positive” if Ct value for Sample (SARS) < 40;

The result is “negative” if Ct value for Sample (SARS) > 45;

The result is “undetermined” if the Ct value for sample (SARS) is between 40 and 45.

**Figure 4:**
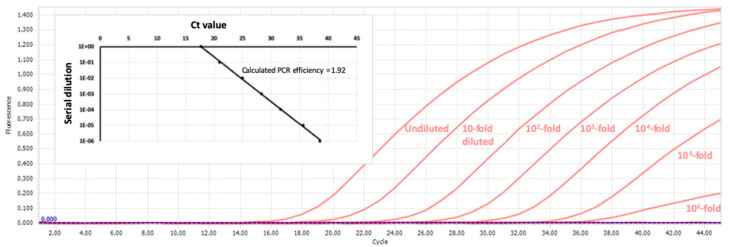
SARS-CoV-2 PCR amplification curves from successive 10-fold dilutions of a positive sample.

The rationale for the Ct cut-off values is based on data generated by serial dilution of positive samples. Figure 4 shows the SARS-CoV-2 PCR amplification curves from successive 10-fold dilutions of a positive sample.

**Figure 5:**
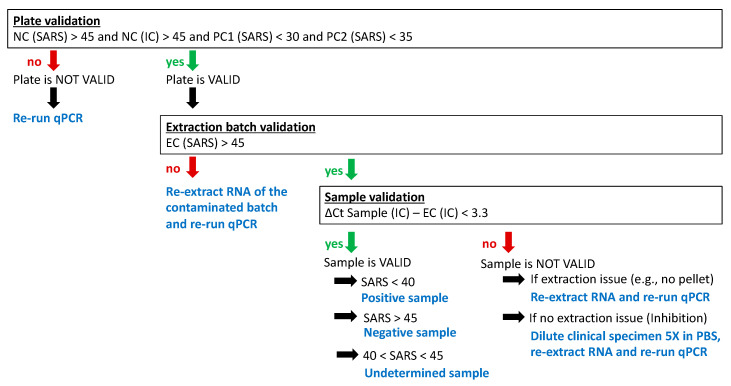
Validation process of the RT-qPCR data.

The validation process is summarized in Figure 5.

Excel files with tracking numbers are used to communicate the results.

If no longer needed, clinical specimens must be autoclaved and discarded appropriately.

## 4. Timing

**Figure 6:**
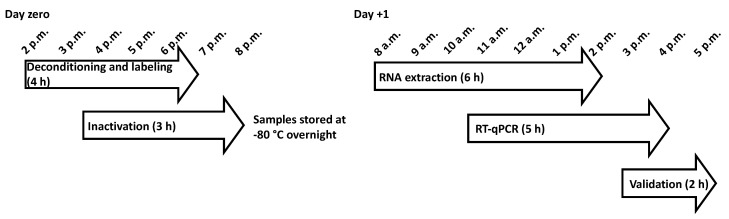
Timeline of the process.

The whole process for the 500 or so samples should be completed in about a day (Figure 6). Samples are received usually at mid-day (day zero) and are deconditioned, labeled, and inactivated by a team of four people. At day +1, RNA extraction should be performed by eight to ten researchers in 6 h. This step requires at least four refrigerated centrifuges (they can be easily shared between researchers). As soon as the first batches of samples are extracted, two to four researchers should start RT-qPCR. They require at least three PCR machines for 5 h, with each machine processing four plates.

## 5. Anticipated Results

**Figure 7:**
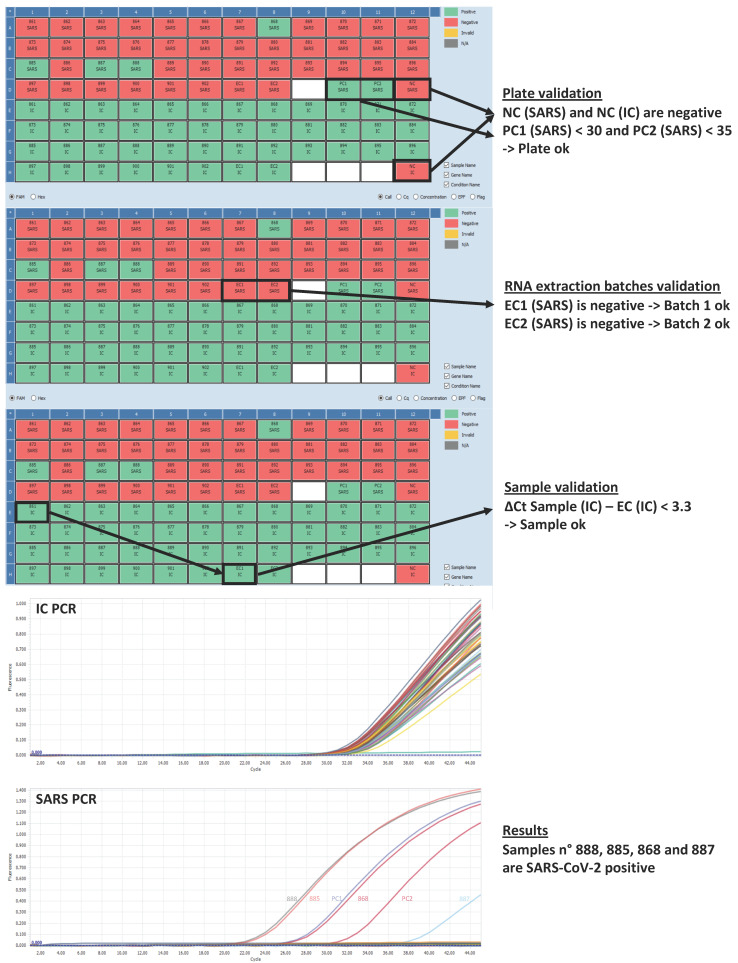
Anticipated results.

Figure 7 illustrates the qPCR data obtained during a typical run. The first steps consist of the validation of the plate, then the validation of the RNA batches, and finally the validation (or the invalidation) of each sample. Supplemental Table S1 contains the raw qPCR data and the spreadsheet for data validation and analysis.

## 6. Notes

1. We do not recommend thermal inactivation of the clinical sample prior to mixing with the solution of guanidinium thiocyanate, as it might lead to false-negative results [4].
2. The present protocol has been tested in dual-color using a FAM probe for the SARS-CoV-2 PCR and a HEX or Cy5 probe for the Internal Control PCR. Unfortunately, the results were not satisfactory, with a strong inhibition of the IC PCR. Therefore, the two PCRs (SARS-CoV-2 and IC) must be done in two separate wells to perform as expected.
3. The primers and Taqman probe targeting the E gene fragment [2] have been preferred to those targeting the RdRp gene. We found the PCR on the E gene to be more sensitive with earlier Ct values on several clinical specimens (data not shown). This might be explained partly by the higher abundance of subgenomic RNAs compared to the genomic ones. The primers and probe binding sequences are conserved among the SARS-related CoV [2]. However, a point mutation on the sequence recognized by the E_Sarbeco_Probe (position 26,340) has been recently reported in some viral isolates, impeding detection [5]. It will be critical to follow the evolution of such strains (https://www.gisaid.org/) and, if necessary, to adapt the protocol using a degenerated probe or targeting a second viral region.
4. In principle, the RNA spike-in used as internal control can be substituted by any known non-human exogenous single stranded RNA.
5. Another alternative to the RNA spike-in control is the titration of house-keeping gene RNA (e.g., Glyceraldehyde-3-Phosphate Dehydrogenase GAPDH mRNA). The advantage of this approach is that it allows viral load comparison between samples. The disadvantage is that it cannot directly identify the samples whose RT-PCR is inhibited and is largely impacted by the type of sample and the sampling technique. Normalization using GAPDH mRNA has been reported for SARS-CoV-1 quantification [6] using the following primers and probes:
  GAPDH-Fw 5′-GAAGGTGAAGGTCGGAGT-3′
  GAPDH-Rv 5′-GAAGATGGTGATGGGATTTC-3′
  GAPDH-Probe 5′-(FAM)CAAGCTTCCCGTTCTCAGCC(BHQ1)-3′
  Further details for house-keeping genes choice and titration can be found in [7,8,9].
6. Because some samples must be redone and others must be fast-tracked, we dedicated an experienced researcher (the trouble-shooter) for that work. They take care of the whole process independently of the other workers to deliver the results in time.
7. Potentially contaminated material (disposable gloves, gown, pipette tips, absorbing paper, FFP2 masks) are autoclaved at 121 °C for 20 min and then incinerated (double-neutralization of highly contagious material).

## Figures and Tables

**Table 1:** Tracking form.

Tracking Form								
								
**Samples processed:**				Date of clinical specimens’ reception:				
								
								
								
**UNPACKING and LABELING**				**Logistician:**				
								
**INACTIVATION**		**Date:**		**Researcher:**				
								
Comments:								
	
								
								
								
								
**EXTRACTION**		**Date:**		**Researcher:**				
								
chemical hood used n°				hood cleaning:				
								
Comments:								
	
								
								
								
								
**RT-qPCR**		**Date:**		**Researcher:**				
								
PCR bench n°				bench cleaning:				
PCR machine n°								
Comments								
	
								
								
								
								
**DATA VALIDATION**		**Date:**		**Researchers:**				
								
Comments								
	
								
								

**Table 2:** Disposition of Samples on the PCR plate.

		1	2	3	4	5	6	7	8	9	10	11	12
**SARS**	**A**	S1	S2	S3	S4	S5	S6	S7	S8	S9	S10	S11	S12
	**B**	S13	S14	S15	S16	S17	S18	S19	S20	S21	S22	S23	S24
	**C**	S25	S26	S27	S28	S29	S30	S31	S32	S33	S34	S35	S36
	**D**	S37	S38	S39	S40	S41	S42	EC1	EC2		PC1	PC2	NC water
**IC**	**E**	S1	S2	S3	S4	S5	S6	S7	S8	S9	S10	S11	S12
	**F**	S13	S14	S15	S16	S17	S18	S19	S20	S21	S22	S23	S24
	**G**	S25	S26	S27	S28	S29	S30	S31	S32	S33	S34	S35	S36
	**H**	S37	S38	S39	S40	S41	S42	EC1	EC2				NC water

**Table 3:** Composition of the SARS-CoV-2 PCR Mix.

Volume in µL	Per Reaction	1 Plate	2 Plates	3 Plates	4 Plates	5 Plates
5X Master Mix	4	200	400	600	800	1000
Euroscript II (RT) and RNAse inhibitor	0.2	10	20	30	40	50
Primers and Probes Mix SARS-CoV-2	4	200	400	600	800	1000
RT Additive	0.2	10	20	30	40	50
RNAse free water	7.6	380	760	1140	1520	1900
Sample or PC or NC or EC	4					

**Table 4:** Composition of the Internal Control (SBV) PCR Mix.

Volume in µL	Per Reaction	1 Plate	2 Plates	3 Plates	4 Plates	5 Plates
5X Master Mix	4	200	400	600	800	1000
Euroscript II (RT) and RNAse inhibitor	0.2	10	20	30	40	50
Primers and Probes Mix IC (SBV)	4	200	400	600	800	1000
RT Additive	0.2	10	20	30	40	50
RNAse free water	7.6	380	760	1140	1520	1900
Sample or PC or NC or EC	4					

## Supplementary Materials

The following are available online at https://www.mdpi.com/2409-9279/3/3/59/s1, Table S1: spreadsheet for analysis and validation of qPCR data.
